# Patient and Healthcare Provider Perspectives on the Implementation of a Web-Based Clinical Communication System for Cancer: A Qualitative Study

**DOI:** 10.3390/curroncol29110662

**Published:** 2022-11-03

**Authors:** Bojana Petrovic, Mary Ann O’Brien, Clare Liddy, Amir Afkham, Sharon F. McGee, Scott C. Morgan, Roanne Segal, Jacqueline L. Bender, Jonathan Sussman, Robin Urquhart, Margaret Fitch, Nancy D. Schneider, Eva Grunfeld

**Affiliations:** 1 Dalla Lana School of Public Health, University of Toronto, Toronto, ON M5T 3M7, Canada; 2 Department of Family and Community Medicine, Temerty Faculty of Medicine, University of Toronto, Toronto, ON M5G 1V7, Canada; 3 Bruyère Research Institute, Ottawa, ON K1N 5C8, Canada; 4 Department of Family Medicine, Faculty of Medicine, University of Ottawa, Ottawa, ON K1G 5Z3, Canada; 5 Ontario Health East, Ottawa, ON K1J 1J8, Canada; 6 The Ottawa Hospital Cancer Centre, Ottawa, ON K1H 8L6, Canada; 7 Department of Radiology, Radiation Oncology and Medical Physics, Faculty of Medicine, University of Ottawa, Ottawa, ON K1N 6N5, Canada; 8 Cancer Rehabilitation and Survivorship, Department of Supportive Care, Princess Margaret Cancer Centre, Toronto, ON M5G 2C4, Canada; 9 Institute of Health Policy, Management and Evaluation, University of Toronto, Toronto, ON M5T 3M7, Canada; 10 Department of Oncology, Faculty of Health Sciences, McMaster University, Hamilton, ON L8V 5C2, Canada; 11 Department of Community Health and Epidemiology, Faculty of Medicine, Dalhousie University, Halifax, NS B3H 1V7, Canada; 12 Department of Surgery, Nova Scotia Health, Halifax, NS B3H 2Y9, Canada; 13 Bloomberg Faculty of Nursing, University of Toronto, Toronto, ON M5T 1P8, Canada; 14 CanIMPACT Patient Advisory Committee, Toronto, ON M5G 1V7, Canada; 15 Ontario Institute for Cancer Research, Toronto, ON M5G 1N8, Canada

**Keywords:** electronic communication, coordination of care, cancer, primary care, qualitative methods

## Abstract

Previous research has identified communication and care coordination problems for patients with cancer. Healthcare providers (HCPs) have reported communication issues due to the incompatibility of electronic medical records (EMR) software and not being consistently copied on patient reports. We evaluated an asynchronous web-based communication system (“eOncoNote”) for primary care providers and cancer specialists to improve cancer care coordination. The objectives were to examine patients’ perceptions of the role of eOncoNote in their healthcare, and HCPs’ experiences of implementing eOncoNote. Qualitative interviews were conducted with patients with breast and prostate cancer, primary care providers, and cancer specialists. Eighteen patients and fourteen HCPs participated. Six themes were identified from the patient interviews focusing on HCP and patient roles related to care coordination and patient awareness of communication among their HCPs. Four themes were identified from HCP interviews related to the context of care coordination and experience with eOncoNote. Both patients and HCPs described the important role patients and caregivers play in care coordination. The results show that patients were often unaware of the communication between their HCPs and assumed they were communicating. HCPs encountered challenges incorporating eOncoNote into their workflow.

## 1. Introduction

Quality cancer care is rooted in providing patient-centred care [1]. According to the Institute of Medicine (IOM), one of the main pillars of patient-centred care is providing care that is integrated and coordinated [1]. Coordination of care refers to “the deliberate organization of patient care activities between two or more participants (including the patient) involved in a patient’s care to facilitate the appropriate delivery of healthcare services” [2]. Previous studies have reported that poor coordination stems from problems with communication between healthcare providers (HCPs) caring for patients with cancer. Cancer specialists and primary care providers (PCPs) have reported issues around not being copied on all reports and challenges resulting from incompatible electronic medical records (EMR) programs, with institutions and clinics using different software to manage patient information [3]. Additional challenges arise from a lack of clarity and communication around roles for various HCPs that are involved in providing cancer care [4,5,6]. These communication issues have negatively impacted patient experience, at times resulting in unnecessary appointments, duplicate services, and higher costs, as well as added stress and confusion for patients [3,5,7,8].

Institutions such as the IOM have called for the implementation of innovations using information technology to support the coordination of care [1]. The use of electronic tools can facilitate the provision of care for patients with complex and chronic conditions such as cancer, who may be receiving care from different HCPs, and in particular when they are transferred between clinical settings (e.g., from primary to secondary care or vice versa) [9]. Text-based clinical communication tools that can be used asynchronously can help avoid challenges associated with traditional methods of communication (e.g., HCPs having to arrange for telephone consultations). Findings from previous studies have demonstrated the benefits of HCPs using asynchronous communication tools, including increased access to specialist consultations, and high satisfaction among HCPs [10,11,12,13].

The present study is part of a broader program of research called the Canadian Team to Improve Community-Based Cancer Care along the Continuum (CanIMPACT), involving a group of researchers, PCPs, cancer specialists, and a Patient Advisory Committee. The overall goal of this program of research was to examine and address gaps in care coordination between oncology and primary care. After conducting several studies to identify gaps in cancer care coordination [3,7,14,15,16,17], the team organized a consultative workshop with stakeholders (patients, caregivers, PCPs, cancer specialists and health system administrators) to obtain recommendations for developing an intervention. Stakeholders recommended implementing a web-based communication system for asynchronous direct communication between PCPs and cancer specialists [18].

Building on our team’s research program, we collaborated with Champlain BASE (Building Access to Specialists through eConsultation) eConsult, a secure web-based system that allows PCPs to directly access specialist advice [19]. The BASE eConsult service was initiated in the Ottawa region of Ontario, Canada, and over 50% of PCPs in this region are registered on this system [20]. We worked with the BASE eConsult team to develop and test a communication system for PCPs and cancer specialists (“eOncoNote”) based on the same platform.

We report here on an embedded qualitative study that was part of a larger mixed methods study of eOncoNote that included a pragmatic randomized controlled trial (pRCT). We sought to answer two research questions: (1) how do patients in the treatment and survivorship phases of the cancer journey perceive the role of eOncoNote in their healthcare; and (2) what are the PCPs’ and cancer specialists’ experiences of implementing eOncoNote in their settings?

## 2. Materials and Methods

### 2.1. Study Design and Intervention

This qualitative study is part of a type 1 hybrid effectiveness-implementation study that used mixed methods. In type 1 hybrid study designs, the primary research aim is to determine the effectiveness of an intervention, and the secondary aim is to obtain a better understanding of the context for implementation [21]. The effectiveness aim was addressed through the pRCT. In this paper, we report on the embedded qualitative study which examines the context of implementation through interviews with patients (examining patient perceptions of their healthcare), and interviews with PCPs and cancer specialists (examining their experience with eOncoNote).

In the larger pRCT, patients were randomized to the intervention group (eOncoNote plus HCPs using usual methods of communication, such as telephone or fax), or control group (usual methods of communication only). For patients randomized to the intervention group, cancer specialists sent an invitation to the patient’s PCP to communicate with them about that patient via the eOncoNote system. The system sent email notifications to a PCP whenever an eOncoNote message was received, prompting them to log into the secure website. Access to the eOncoNote system was made available to cancer specialists and PCPs whose patients consented to take part in the study. The duration of access to the eOncoNote system depended on the patient’s phase of cancer care (treatment or survivorship) and disease site (breast or prostate cancer). For patients receiving treatment for prostate cancer or breast cancer, PCPs and cancer specialists were able to communicate via eOncoNote for 4 months or 6 months, respectively. For patients with breast cancer who were transferred to survivorship care, PCPs and cancer specialists were able to communicate via eOncoNote for 1-year post-transfer to primary care. At the cancer centre where recruitment took place, it is usual practice for breast cancer survivors to be transferred back to their PCP through a nurse-led survivorship program following the completion of their treatment [22]. Breast and prostate cancer were selected as they are among the most common types of cancer in Canada [23]. We focused on these disease sites for feasibility purposes, given that this was the first time that eOncoNote system was being implemented.

### 2.2. Conceptual Framework

This study was informed by constructivist grounded theory principles [24]. The main purpose of grounded theory is to generate explanatory models grounded in the data, and it involves simultaneous data collection and analysis [25]. With the constructivist grounded theory approach, the qualitative interview serves as the place for engaging the researcher and participant in the co-construction of knowledge, including the meanings that the researcher observes [26].

This study draws on the Consolidated Framework for Implementation Research (CFIR) [27]. The CFIR framework comprises five major domains: intervention characteristics, outer setting, inner setting, characteristics of individuals, and implementation process [27]. As a determinant and multi-level framework, the CFIR describes factors from the individual to the organizational level hypothesized to influence implementation outcomes but does not specify causal mechanisms [28]. The CFIR informed the development of the interview guide for HCPs.

### 2.3. Research Ethics

Approval was obtained from two research ethics boards, the Ottawa Health Science Network Research Ethics Board and the Health Sciences Research Ethics Board at the University of Toronto.

### 2.4. Sample and Recruitment

#### 2.4.1. Patient Sample

A purposeful sample [29] of patients was recruited to participate in interviews to gather perspectives from patients in different age groups and cancer types. Two groups of patients randomized to the intervention group of the eOncoNote pRCT were recruited: (a) patients on active treatment for breast or prostate cancer, and (b) patients who had completed treatment for breast cancer and were part of a survivorship program involving transfer to their PCP (see Table 1 for patient inclusion and exclusion criteria).

#### 2.4.2. HCP Sample

A purposeful sample [29] of HCPs, including PCPs and cancer specialists who participated in the pRCT, was recruited to participate in interviews. Cancer specialists included medical and radiation oncologists, program managers, and nurses from the survivorship program.

#### 2.4.3. Recruitment of Patients

**Figure 1:**
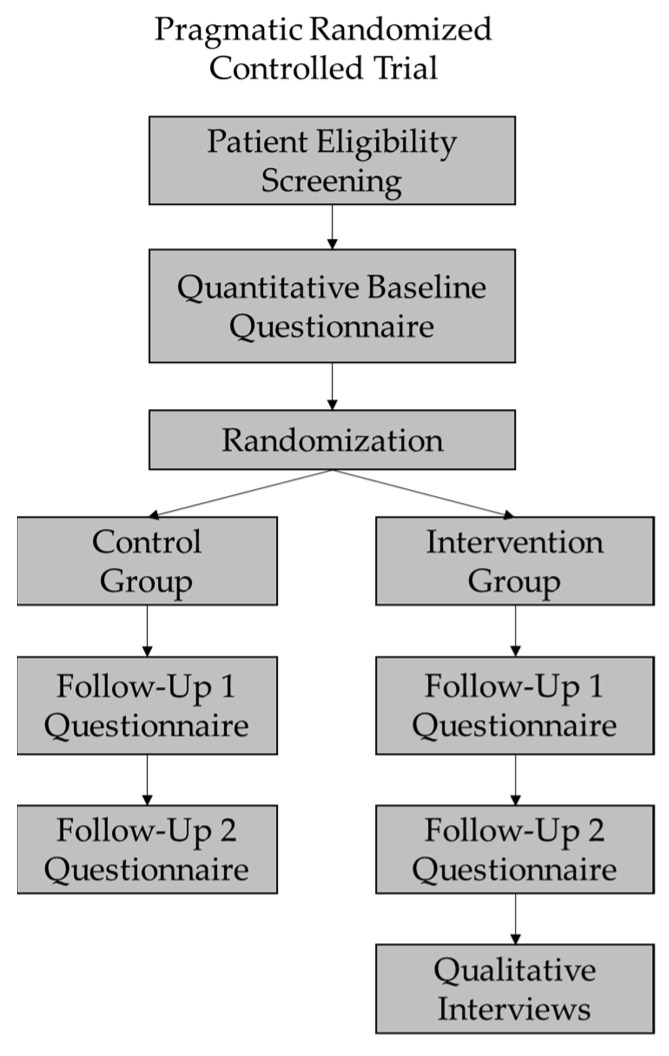
Sequence of patient quantitative and qualitative data collection.

Patient recruitment took place at a large academic hospital cancer centre in Ontario, Canada where the pRCT was conducted. Patient participants who met the inclusion criteria for the trial (Table 1) were identified by the cancer specialists and invited to meet with a research assistant (RA) to learn about the study. At the time of providing informed consent for the pRCT, patients were asked if they were potentially interested in participating in a qualitative interview upon completing the trial (see Figure 1). Patients who were randomized to the intervention group and expressed interest in an interview were contacted to arrange a date and time for the interview. Recruitment for the overall study (pRCT and qualitative interviews) began in February 2018 and ended in March 2020.

#### 2.4.4. Recruitment of HCPs

PCPs were invited by the RA via email to participate in an interview. Cancer specialists were also invited by the RA via email to participate at the end of the study. Some cancer specialists were interviewed before the end of the study to accommodate staffing changes (e.g., moving on to other roles at the hospital). In addition, study co-investigators sent two emails to each HCP to invite them to participate in interviews at the end of the study.

### 2.5. Data Collection

Semi-structured telephone interviews were conducted by BP (MPH; Research Associate and PhD candidate; female) using interview guides. The interview guides were developed in consultation with our HCP research team members and the CanIMPACT Patient Advisory Committee. Interviews were conducted by telephone to accommodate participants’ availability, minimize the burden, decrease travel costs, and increase access to respondents across a wide geographic area. Patient interviews focused on examining perceptions of communication and coordination between PCPs and cancer specialists and the role of eOncoNote in their healthcare. Interviews with HCPs examined barriers and facilitators to implementing eOncoNote into existing workflows.

### 2.6. Data Analysis

Interview recordings were transcribed verbatim. In following the constructivist grounded theory, the constant comparative method was used to analyze the interview transcripts [30]. NVivo 12 software [31] was used for data management. Following Boeije, ref. [32] comparisons were made within and between interviews, and between different groups of participants (e.g., HCPs and patients). Initial coding was completed by two team members (BP and MAO) using open coding (i.e., the central codes that emerged were derived from the data, rather than being pre-determined). Coding guides were developed based on the initial analysis of 9–10 transcripts (approximately 3 for each of the patients, PCPs, and cancer specialists per Fonteyn et al. [33]). After consensus was reached between BP and MAO, the coding guides were applied by BP for the remainder of the transcripts and revised periodically. Although CFIR informed the interview guide development, we did not use CFIR in the data analysis to avoid using pre-determined coding categories.

## 3. Results

### 3.1. Participants

Of the 25 patients approached for an interview, 18 patients participated, including six patients receiving treatment for breast cancer, six patients receiving treatment for prostate cancer, and six breast cancer survivors who were transferred back to their PCP after completing treatment as part of the cancer centre’s survivorship program. Fourteen cancer specialists were approached for a telephone interview; 12 participated, including one program manager, two oncology nurses, and nine oncologists. Of the 61 PCPs that were approached, two agreed to participate. Table 2 presents participant characteristics. Patient interviews ranged from 16 to 81 min in duration (average of 42 min), and HCP interviews lasted between 21 and 47 min (average of 34 min).

### 3.2. Themes

Although the research question focused on the role of eOncoNote on patient perceptions of their healthcare, patients also described the context of their cancer care experiences and perceptions regarding HCP communication in general. Six themes were identified describing HCP and patient roles related to cancer care coordination, and patient awareness of the communication occurring among their HCPs. Four themes were identified in the HCP interviews related to the context of cancer care coordination and experience with communication via eOncoNote.

#### 3.2.1. Patient Interviews

Theme 1: Primary care providers were mainly involved in providing care during the diagnosis and survivorship phases.

Patients described their PCPs’ as being more involved during the diagnosis and survivorship phases of cancer care, and less involved during treatment. Patients who saw their PCP during the treatment phase spoke about their involvement in managing comorbid conditions and providing mental health support.

“When I did call [PCP], he says as long as you’re followed [during treatment phase], you feel that you’re being followed properly by the oncologist, I didn’t have to go to him unless I really needed to. [PCP is] actually the one who scolded me into going to get a mammogram…then he got the results and that’s how it all started.”

(P4)

Theme 2: Patients described the value of feeling connected with their HCPs.

When asked about their HCPs’ involvement in cancer care, patients often described interactions in terms of what and how PCPs and cancer specialists communicated with them. Patients appreciated when HCPs showed empathy, when they felt connected with their HCPs (e.g., by discussing their interests and family life), and when they received information specific to their unique circumstances.

“My GP, she tells me the facts but it’s always very human, compassion, we’re going to find a solution, you know? So you don’t leave completely startled, thinking okay, now my fear is back, I’m going to die.”

(P6)

Theme 3: Patients’ experiences with cancer care were influenced by social factors.

Patients valued sharing their experience with family members or friends who had cancer, and at times reflected on the services their loved ones received compared to their own care. They described the importance of social support, such as having family members attend appointments with them. Having flexible work arrangements while receiving cancer treatment and being able to take sick days made a positive impact on their cancer care experience. Conversely, patients who did not have flexible work schedules reported challenges with coordinating their healthcare appointments.

“I had bought a house during this process, and we moved while I was having chemo. And I didn’t want to go on [income support for unemployed workers]. I wanted to keep having my paycheque because we were already starting the process of a house before I got the diagnosis. And so I focused on my kids and my work. And there were days I couldn’t work. There were days I was very sick. And my employers were great…They were super accommodating…My boss said, “Just send me a text if there’s any days you can’t do it. Just say I can’t do it…” No questions asked. So that was really nice. And that helped me keep working.”

(P13)

Theme 4: Patients are often involved in their own cancer care coordination.

Patients described that during their cancer journey, they were often involved in coordinating their own cancer care. Examples of patients’ roles in coordination included transferring information from one HCP to another, making sure that PCPs were informed about their survivorship care plan and advocating for tests as part of their follow-up care. Patients recognized that it was important for their PCP to be informed about their cancer care, checking whether their PCP was copied on test results and whether they received survivorship care plans. While some patients accepted this role as another step they needed to complete, others reported feeling awkward about having to be proactive and ask for certain types of follow-up (e.g., physical exams for breast cancer follow-up).

“[PCP] wanted me to keep her in the loop what was going on with everything. She says that once I found out stuff I was supposed to call and tell her, the results and everything.”

(P2)

Theme 5: Patients are often unaware of the communication among HCPs.

When asked about how well their PCP and cancer specialist worked together, many patients were unaware of their collaboration or communication. Some patients felt they could not answer because they had not seen their PCP during treatment, while others assumed that their HCPs were communicating because they did not notice any indication of HCP communication problems. Many of the patients did not know that their PCPs had access to eOncoNote. Patients who were aware assumed that their PCP had access because the patient had consented to participate in the research study, and they did not know how their PCP’s access to the system could have impacted their cancer care.

“It’s difficult for me to answer [how well PCP and radiation oncologist worked together], as I say, because I have not had contact with my family physician. I’m assuming that the information was passed on.”

(P14)

Theme 6: Patients have differing levels of comfort with being involved in a web-based discussion with HCPs.

Although eOncoNote was designed to be a one-to-one communication system between PCPs and cancer specialists, our Patient Advisory Committee advised us to ask patients about their interest in potentially being involved in a discussion with HCPs via a web-based system. Some patients were in favour of participating in a web-based text discussion with their PCP and cancer specialist, citing that it could be helpful to get questions answered that they may have forgotten to ask during their appointment or generate questions for their future appointments. However, others were not comfortable with this, raising concerns that viewing discussions between HCPs online might be anxiety-provoking and could lead to misunderstanding about medical terminology.

“I mean really the only way I can see that a patient could be involved in it is to have an area maybe where they could… put down if they do have any concerns. But I’m hesitant to say that because I did have access… Through the [hospital], I did have access to a lot of information on the tests that I had done and files…And you know, I would look at those lab reports and I can’t interpret them. I’m not an expert… I need an expert to sit down with me and do that… I mean I think everybody wants everybody to get online and stuff. But it’s not necessarily the best thing. I’m not sure that we as patients are able to deal with that kind of information.”

(P10)

#### 3.2.2. HCP Interviews

Theme 1: There was limited communication between cancer specialists and PCPs while patients received active treatment.

Overall, cancer specialists reported infrequent communication with PCPs while the patient was receiving active treatment. Some oncologists felt that the patient’s cancer care during the treatment phase was their responsibility, while others hypothesized that the limited communication was because PCPs were busy or may not have felt comfortable addressing concerns if they had limited training in oncology. This sentiment was echoed by the PCPs, noting that they were not involved as much once the patient entered the cancer system, feeling like an “outsider” and getting updates along the way. Most communication between cancer specialists and PCPs outside of the hospital relied on using fax. As such, many cancer specialists described feeling like their usual consult notes were being “sent into a vacuum” because they often did not hear back from PCPs or have a confirmation that their note was received.

“Primary care, I feel it’s more of a one-way communication. Like I know our notes go to them. But it’s rare that, you know, it’s proper communication or I hear something back from a physician. It’ll be very rare for me to have that.”

(HCP 11)

Some cancer specialists were nervous about this and took extra measures to call the PCPs to ensure that an issue identified in their note was going to be addressed. Communication with PCPs was more frequent for patients with complex needs (i.e., if they had comorbid conditions including mental health issues).

“I think the biggest ones that I’ve had back and forth have tended to be either patients with more comorbid conditions, so more issues, more non-cancer issues that require management…And the other scenario would be patients who have pre-existing, particularly psychiatric or just anxiety or aspects of their care, insomnia, things that probably are chronic and have a back story to. So like how the patient responded to different treatments or therapies. That is hard as someone who doesn’t know the patient quite as well. Like many of the family doctors have been taking care of the patients for years.”

(HCP 10)

After the patient completed treatment and was transferred to primary care, cancer specialists reported that PCPs communicated with them regarding endocrine therapy and concerns about starting new medications or cancer recurrence. Several cancer specialists reported more involvement from PCPs whose patients lived far from the cancer centre.

Theme 2: Nurses and clerical staff, and patients and caregivers help to facilitate cancer care coordination.

When asked about facilitators to care coordination, HCPs discussed the involvement of nurses and clerical staff, as well as patients and caregivers. HCPs described how nurses and clerical staff helped to facilitate cancer care coordination, including facilitating communication with other specialists (e.g., surgeons) and communicating with PCPs if they had concerns after a patient was transferred back to primary care. Nurses in the survivorship program helped to systematically gather patient information from different oncologists and compile it into a standardized report for PCPs.

“Talking about the [survivorship] program, I find that’s a very neat program we have here where there’s a proper documented explanation of exactly how the patient was treated for their cancer, and that copy is sent to the primary care physician highlighting what kind of surveillance they require. I think in the absence of that program, like I don’t think we would be doing a good job, per se, in handing over all the information that the primary care physician would need to know for a cancer patient… [Survivorship program] gathers the data from all of the oncologists and sends one report to the primary care physician.”

(HCP 11)

HCPs also described the important role that patients and caregivers play in coordinating cancer care. Examples of patients’ involvement in care coordination included providing updates about medication changes and relaying test results from one HCP to another. HCPs noted that having a point person (such as the patient or family member) who knows what is happening at all times makes cancer care coordination flow more seamlessly.

“As much as I think the health care system needs to maybe make it easier, I think at the same time a lot of good facilitation comes from the patient end. So, when there is that maybe patient advocate, whether it’s a family member or daughter or son or spouse… You know, the person carrying the binder and knows exactly what’s happening at all times. Things do happen a bit more seamlessly because they’re sort of on point and they know exactly what’s happening at each point. I don’t know how that would potentially be integrated and maybe rolled out at a wider level. But it does facilitate things happening seamlessly.”

(HCP 12)

Theme 3: Barriers to cancer care coordination included relying on traditional methods of communication and a lack of access to the same electronic medical record (EMR) among HCPs.

When asked about barriers to care coordination, HCPs described challenges with traditional methods of communication (e.g., telephone and fax), including leaving voicemails and playing “phone tag” with other HCPs.

“You’d have to leave messages on voicemail, and voicemail wouldn’t accept messages. So you had to call back many, many times. So actually getting the professional you wanted was commonly a challenge.”

(HCP 2)

The lack of access to an integrated EMR system across the province was reported as another major challenge. Not having the ability to access the same clinical information required HCPs to rely on patients to relay information between HCPs. Cancer specialists described features within the newly implemented Epic EMR system [34] that facilitated communication with colleagues who were part of the same system, including internal email and instant messaging. Cancer specialists discussed these features in contrast to the traditional communication methods they use with physicians in the community (including PCPs and specialists practicing outside the hospital).

When asked about challenges with regard to implementing eOncoNote, cancer specialists and PCPs reported concerns about it being a separate system requiring them to log in with a password that would expire and need to be continuously updated. HCPs recommended creating a solution to integrate eOncoNote into their EMR. Since eOncoNote only allowed for one-to-one communication, HCPs felt that having EMR integration could help address concerns around access to patient information if an HCP was on leave and another clinician needed to access the information in their absence.

“The government in Ontario should just invest in [single EMR system]… It would be so helpful in saving money, avoiding duplication of tests, improved communication. There are so many players now involved in e-health—from the [Ontario Telemedicine Network] telemedicine networks and, private companies. They’re just fragmenting something that really should be unified. And every family doctor shouldn’t have to pay thousands for their own EMR that doesn’t talk to others. That really should be the logical way to go.”

(HCP 7)

Theme 4: eOncoNote had the potential to be a useful tool but it was not used extensively.

HCPs reported that although eOncoNote had the potential to fill a gap by offering a way to electronically communicate with their colleagues, they noted that it was not used as much as they expected. In many patient cases, cancer specialists indicated that PCPs did not respond to their messages or send questions via eOncoNote. Since PCPs were still receiving faxed clinical notes, and there were few questions coming in via eOncoNote, some cancer specialists questioned whether it would be valuable to continue sending PCPs invitations to communicate for all patients.

“So I think [eOncoNote] functioned well and for what it did. But I must say that I didn’t have a lot of messages coming in from the family physician. And of course, in parallel, we always had the clinic notes that were being sent through another system, being sent to the primary care provider…And I just think for most patients, that was probably sufficient.”

(HCP 4)

Cancer specialists described that one of the common concerns among colleagues at the outset was whether eOncoNote was going to create an additional workload. They became more comfortable with using the eOncoNote system as they had more of their patients participating in the pRCT, allowing them to familiarize themselves with the eOncoNote system and the communication process. Some participants said that new workflow changes at the cancer centre had resulted in additional administrative responsibilities for oncologists with little time left to learn a new system. Cancer specialists emphasized the need to have a unified approach to communication with PCPs, without having to access multiple communication platforms. They felt that broader uptake by PCPs may help reduce the fragmentation of care.

## 4. Discussion

This study examined patient and HCPs’ perspectives on the implementation of a web-based, two-way, asynchronous communication system ‘eOncoNote’, how patients perceived the role of eOncoNote in their healthcare, and the HCPs’ experiences of incorporating it into their clinical practice. Results indicate that, although eOncoNote was designed to fill a perceived gap in HCPs’ ability to communicate, it was not used as much as expected. The infrequent use of the eOncoNote system may be due in part to the trial study design, as each PCP could only have one patient enrolled in the trial to control for contamination between intervention groups. Previous studies have found that longer-term use of electronic tools positively influences adoption attitudes among PCPs [35]. Perhaps more opportunities to use the eOncoNote system, with more patients, could have led to greater adoption among PCPs. Alternatively, there may have been less need among PCPs and cancer specialists for web-based communication regarding this particular patient population. For example, we learned that in most cases, PCPs communicated infrequently with cancer specialists regarding patients on active treatment. Findings from Easley and colleagues’ [4] suggest that PCPs have limited knowledge and training opportunities regarding cancer care. In this study, PCPs were more likely to contact cancer specialists by telephone or fax after the patient completed treatment, in instances where a patient had more complex needs, or if the patient lived far away from the cancer centre. During the study, the participating cancer centre implemented the Epic EMR system [34] which replaced several separate systems that were previously used; however, cancer specialists reported that communication with PCPs was mainly conducted via fax as only a minority of PCPs in the region had access to Epic. Traditionally, communication was carried out one way with clinical notes being sent from cancer specialists to PCPs. HCPs stressed the need to create a unified solution for communication, which should be integrated with both the PCPs’ and hospital’s EMR systems to help avoid the challenges of traditional communication methods. Implementing integrated EMR systems would allow HCPs to track and share information about their patients’ symptoms, preferences regarding care and health outcomes [5,36].

During interviews with patients, participants shared that their PCPs were involved in providing care primarily while they were undergoing diagnostic investigations and then after they completed treatment and were transferred from the cancer centre. However, results from administrative health data studies in four Canadian provinces show that patients with breast cancer were seen more often by PCPs during the treatment phase than pre-diagnosis, and were more likely to be high users of primary care with a greater number of comorbidities [17]. Perhaps the participants in this study were less complex patients with fewer comorbid conditions and as such, did not require extensive PCP involvement. Another theme that emerged in this study was that patients valued feeling connected to their HCPs through their cancer journey, noting the importance of sharing about their family life and personal interests. Similar to previous research [7], patients in this study valued the way HCPs communicated with them (i.e., showing empathy, being careful with the choice of words they use to describe the patient’s cancer care, etc.). Patients shared that they often did not know about the communication occurring among their HCPs and assumed they were communicating unless a specific problem arose that suggested otherwise. When asked about their potential interest in participating in an online discussion with their HCPs, some patients expressed interest in the idea of being able to communicate with their HCPs via a web-based system, while others said that the online discussion between their providers might be difficult to understand and anxiety-provoking. Patients’ perceptions about participating in online communication with HCPs may have been influenced by their experience of accessing patient portals, as some commented on having reviewed their records and clinical notes through the hospital patient portal, MyChart [37].

Comparing the results of the two groups of participants (HCPs and patients), we found alignment on several themes. First, cancer specialists described limited communication with PCPs while patients received treatment, which was consistent with patient reports that their PCPs were primarily involved in providing care during diagnosis and post-treatment follow-up. Second, patients reported that they were unaware of the communication occurring between their HCPs, and most did not know their PCP had access to eOncoNote. Patients may have been unaware of the communication because, as cancer specialists reported, PCPs did not send many messages via eOncoNote. The strongest alignment between the patient and HCP interviews was related to the role of patients and caregivers in coordinating cancer care. Both patients and HCPs provided examples of patient and caregiver involvement in coordinating cancer care, from transferring information from one HCP to another, to making sure their cancer surveillance tests were scheduled. While some patients seemed to have accepted this role as part of their cancer journey, others felt less comfortable in situations where they had to request follow-up tests or physical exams. Other studies examining patient perspectives on their role in coordinating care have reported problems with this role, including challenges with navigating the healthcare system, feeling the burden of taking on the role of “project manager” to coordinate their own care, and difficulty bringing up needs for additional support [7,38].

This qualitative study provided patient perspectives on cancer care coordination, communication between HCPs, and the role of eOncoNote in their healthcare. The study also offered insights into the barriers and facilitators of implementing eOncoNote within the cancer centre of an academic hospital. Additional strengths of this research include developing interview questions based on CFIR and using an inductive approach to code interview transcripts. With this approach, central codes and emerging themes were derived from the data, rather than being pre-determined and limited to the constructs specific to CFIR.

Several limitations should be noted. The study sample was self-selected, and thus it is possible that HCPs who experienced more challenges with using eOncoNote or patients who had more negative experiences with their HCP decided not to participate. Moreover, cancer specialists reported that PCPs did not communicate extensively with them via eOncoNote, and only two PCPs agreed to participate in a qualitative interview. Thus, we did not reach thematic saturation of the PCP interviews. Perhaps PCPs did not respond to invitations for a qualitative interview because they felt they did not have much feedback to share due to their limited experience with eOncoNote and the fact that they only had one patient that was part of the study. Previous studies have reported barriers to PCP participation in research, including having other obligations, not having enough time to participate in research, and a perceived lack of relevance to their clinical work [39,40].

## 5. Conclusions

This study examined the experiences of implementing eOncoNote from multiple perspectives (different types of HCPs and patients from treatment and survivorship phases of cancer care). Patients are often unaware of the communication between their HCPs and assume that they are communicating unless problems arise. While some patients may accept the role of coordinating their cancer care, others may not be comfortable doing so. Clinicians should assess patient readiness to take on the cancer coordination role across the patient’s cancer journey. Future research should examine how communication systems could be better adapted and integrated into existing EMR software across primary care and hospital systems. In addition, provincially implemented EMR solutions [41] may help to address HCPs’ concerns around access to patient information. Multijurisdictional research teams should examine whether having access to provincial EMR software could help facilitate the coordination of care for patients with cancer.

## Figures and Tables

**Table 1:** Patient inclusion and exclusion criteria.

Inclusion Criteria	Exclusion Criteria
At least 18 years old No prior history of cancer in the past 5 years (those with non-melanoma skin cancer could participate) Receiving adjuvant chemotherapy for early-stage breast cancer, or radical or adjuvant radiation therapy for localized prostate cancer; or completed adjuvant therapy for breast cancer and participating in the survivorship program with the intent of being transferred for survivorship follow-up care to their PCP.	Participating in another study requiring ongoing questionnaire completion Does not have a PCP Their PCP has another patient enrolled in the trial Unable to read and write in English, and Unable to provide informed consent.

**Table 2:** Participant characteristics.

Participant Role	Characteristic	*n* (%)
Patient (*n* = 18)	Age (years)	
	30–49	3 (17)
	50–59	5 (28)
	60–69	6 (33)
	70+	4 (22)
	Sex	
	Female	12 (67)
	Male	6 (33)
	Type of cancer	
	Breast	12 (67)
	Prostate	6 (33)
	Phase of cancer care	
	Treatment	12 (67)
	Survivorship	6 (33)
Healthcare Provider (*n* = 14)	Age (years)	
	30–39	6 (42)
	40–49	2 (14)
	50–59	3 (21)
	Not available	3 (21)
	Sex	
	Female	6 (43)
	Male	5 (36)
	Not available	3 (21)
	Type of provider	
	Cancer program manager	1 (7)
	Oncology nurse	2 (14)
	Oncologist	9 (64)
	PCP	2 (14)
	Years in practice	
	1–9	6 (43)
	10–19	2 (14)
	20+	3 (21)
	Not available	3 (21)

## Data Availability

Contact the corresponding author.
